# Analysis of an ordinal outcome in a multicentric randomized controlled trial: application to a 3- arm anti- malarial drug trial in Cameroon

**DOI:** 10.1186/1471-2288-10-58

**Published:** 2010-06-18

**Authors:** Solange Youdom Whegang, Leonardo K Basco, Henri Gwét, Jean-Christophe Thalabard

**Affiliations:** 1 Université de Yaoundé I, Ecole Nationale Supérieure Polytechnique, Département de Mathématiques et Sciences Physiques, B. P. 8390, Yaounde, Cameroon; 2 Université Paris Descartes, Laboratoire de Mathématiques Appliquées (MAP5), Unité Mixte de Recherche 8145, Centre National de la Recherche Scientifique, 45 rue des Saints Pères, 75006 Paris, France; 3 Université Aix-Marseilles 2, Unité de recherche sur les maladies infectieuses et tropicales émergentes (URMITE). Unité Mixte de Recherche 198-Institut de recherche pour le développement. Faculté de Médecine la Timone, 27 boulevard Jean Moulin, 13385 Marseille Cedex 5, France

## Abstract

**Background:**

Malaria remains a burden in Sub-Saharan Countries. The strategy proposed by the World Health Organization (WHO) is to systematically compare the therapeutic efficacy of antimalarial drugs using as primary outcome for efficacy, a four-category ordered criterion. The objective of the present work was to analyze the treatment effects on this primary outcome taking into account both a center-effect and individual covariates. A three-arm, three-centre trial of Amodiaquine (AQ), sulfadoxine-pyrimethamine (SP) and their combination (AQ + SP), conducted by OCEAC-IRD in 2003, in 538 children with uncomplicated *Plasmodium falciparum *malaria, is used as an illustration.

**Methods:**

Analyses were based on ordinal regression methods, assuming an underlying continuous latent variable, using either the proportional odds (PO) or the proportional hazards (PH) models. Different algorithms, corresponding to both frequentist- and bayesian-approaches, were implemented using the freely available softwares R and Winbugs, respectively. The performances of the different methods were evaluated on a simulated data set, and then they were applied on the trial data set.

**Results:**

Good coverage probability and type-1 error for the treatment effect were achieved. When the methods were applied on the trial data set, results highlighted a significance decrease of SP efficacy when compared to AQ (PO, odds ratio [OR] 0.14, 95% confidence interval [CI] 0.04-0.57; hazard ratio [HR] 0.605, 95% CI 0.42-0.82), and an equal effectiveness between AQ + SP and AQ (PO, odds ratio [OR] 1.70, 95% confidence interval [CI] 0.25-11.44; hazard ratio [HR] 1.40, 95% CI 0.88-2.18). The body temperature was significantly related to the responses. The patient weights were marginally associated to the clinical response.

**Conclusion:**

The proposed analyses, based on usual statistical packages, appeared adapted to take into account the full information contained in the four categorical outcome in malaria trials, as defined by WHO, with the possibility of adjusting on individual and global covariates.

## Background

Many clinical trials have a primary outcome which is measured on a categorical scale. In these situations, the outcome categories are often ordered, either by a direct relationship to either a continuous measurement or a clinical score through pre-defined cut-points, or by a natural interpretation by the physician of a health condition or body reaction.

An overview and recent developments in the analysis of ordered categorical data have been presented in [1-4]. More specific developments for analyzing ordinal data in the context of meta-analysis of clinical trials have been proposed initially for summary statistics [5,6], secondarily extended to meta-analysis with individual patient's data [7,8].

The main objective of this paper was to adapt and implement the above-proposed methods in the specific context of malaria. A more specific objective was to study the effect of both different treatments and a set of covariates on the primary outcome.

In antimalarial trials, the WHO (World Health Organization) established a clinical study protocol [9,10] to assess the efficacy of antimalarial drugs for uncomplicated malaria, in which the primary outcome is a four-category outcome, i) ACPR (*adequate clinical and parasitological response*); ii) LPF (*late parasitological failure*); iii) LCF (*late clinical failure*) and; iv) ETF (*early treatment failure*). Patient status reflects the ordinal character of the WHO criteria. Danger signs appearing on day 1, 2 or 3 with a persistent parasitaemia correspond to an ETF. The presence of fever and parasitaemia between day 4 and day 14 without an ETF is considered as LCF, whereas the presence of parasitaemia on day 14 without ETF and LCF is a LPF. ACPR corresponds to the absence of parasite regardless the body temperature on day 14 without being classified as either ETF, or LCF or a LPF previously. This suggests that there is an explicit order between the categories. A multi-centre randomized controlled trial of three antimalarial drugs, amodiaquine (AQ), sulfadoxine-pyrimethamine (SP) and their combination (AQ + SP), which was conducted in 2003 by OCEAC-IRD in patients with uncomplicated *P. falciparum *malaria from 3 health centers in Cameroon, according to the WHO protocol [9,10] will be used to illustrate the statistical approach.

Briefly, the 3 centers were selected as they were known to be associated with different levels of transmission. Affected children, 0-5 years old, were the target population, according to the WHO recommendations for assessing antimalarial drug efficacy. The primary outcome of efficacy was the four category-variable: C_1 _= ACPR, C_2 _= LPF, C_3 _= LCF, and C_4 _= ETF, the first category being the best category and the last category being the worst, in terms of health condition. Table 1 describes the results for each arm of anti-malarial drug, in the three centers. The majority of patient outcomes were assigned in the first category, with very few treatment outcomes in the other categories, corresponding to various degrees of therapeutic failures. The protocol was approved by both the Ministry of Public Health and the Ethical National Committee. The person legally in charge of the children gave an informed consent before inclusion in the study. Study investigators were OCEAC field workers. A global analysis of the data, based on the rate of ACPR can be found in [11].

**Table 1:** Counts of treatment response by category stratified in all centers and treatments.

		C_1_	C_2_	C_3_	C_4_	
Center	Treatment	ACPR	LPF	LCF	ETF	Total
	AQ	62	0	0	1	63
		[98.4]**	[0]	[0]	[1.6]	[100.0]
1	SP	53	2	0	6	61
		[86.8]	[3.27]	[0]	[9.8]	[100.0]
	AQ+SP	59	0	0	0	59
		[100.0]	[0]	[0]	[0]	[100.0]
	AQ	52	2	0	0	54
		[96.3]	[3.7]	[0]	[0]	[100.0]
2	SP	48	0	2	3	53
		[90.56]	[0]	[3.7]	[5.26]	[100.0]
	AQ+SP	55	1	0	0	56
		[98.2]	[1.8]	[0]	[0]	[100.0]
	AQ	57	0	0	1	58
		[98.2]	[0]	[0]	[1.7]	[100.0]
3	SP	52	2	0	3	57
		[91.2]	[3.5]	[0]	[5.26]	[100.0]
	AQ+SP	58	0	0	0	58
		[100.0]	[0]	[0]	[0]	[100.0]
						

	Total	496	7	2	14	519
		[95.5]	[1.3]	[0.38]	[2.7]	[100.0]

** Row percentage. The categories C_1_, C_2_, C_3_, and C_4 _represent ACPR, LPF, LCF and ETF, respectively.

## Methods

### Statistical methods

#### The proportional odds model

The statistical approach is based on the proportional odds model [12]. Consider the *K *= 3 centers. Each patient had a response into one of the *m *= 4 categories, (*C*_1_, .., *C*_*m*_), with *n*_*i *_patients in center *i*. Let  be the total number of patients overall.

Denote *Z *the response and *π*_*ijc*_, the probability that the *jth *subject in study *i *had a response in category *c*; *π*_*ijc *_= Pr(*Z*_*ij *_= *c*). Let also *Q*_*ijc *_= Pr(*Z*_*ij *_≤ *c*) be the cumulative probability of a response in category *c *or better, so that *Q*_*ijc *_= *π*_*ij*__1 _+ ... + *π*_*ijc *_and *Q*_*ijm *_= 1; *c *= 1, ..., *m*-1. Hence *c *= *m *corresponding to the worst outcome (here an ETF) is the baseline level. The proportional odds model is defined by(1)

where *α*_*c *_represents the *cth *intercept and *η*_*ij *_= *γ*_1 _*x*_1__*ij *_+ ...+ *γ*_*p *_*x*_*pij*_, is a linear combination of explanatory variables. The model assumes 1) an increasing order for the *α*_*c*_: *α*_1 _<*α*_2 _< ... <*α*_*m*-1_; 2) *α*_0 _= -∞ and *α*_*m *_= ∞.

The assumption of proportional odds signifies that the ratio of the odds of the event *Z*_*ij *_≤ *c *for any pair of sets of explanatory variables is independent of the category *c*. The cumulative logit model (1) results from the existence of a latent random variable with a logistic distribution, the (*α*_*c*_, c = 0, ..., m) corresponding to the cut-off thresholds for categorizing the latent variable into *m *classes. Alternatives to the proportional odds model can be developed based on different assumptions regarding the latent variable, like a normal- or an extreme value distribution associated with the probit or complementary log- log links, respectively.

##### Fixed effect model

The basic model extends the proportional odds model (PO) proposed in [7] to a situation with L treatments (L > 2). For the sake of simplicity, the presentation will be restricted to the situation of L = 3 treatments corresponding to the real data set but the extension to more than 3 treatments is straightforward. Assuming that one particular treatment (here, AQ) was selected as the reference treatment, the treatment covariate is modeled using L-1 = 2 dummy variables *T*_1 _and *T*_2_, where *T*_*l *_(l = 1, L-1) yields 1 if subject received the treatment l, and 0 otherwise (here, l = 1 corresponds to SP, l = 2 corresponds to AQ + SP)(2)

where {*j *= 1, ..., *n*_*i*_,} corresponds to the individuals within each arm, {*i *= 1, .., *K*}, corresponds to the centers and {*c *= 1, ..., *m*-1} to the categories. The parameter *γ*_1 _represents the log-odds ratio of efficacy associated to treatment 1 *versus *the reference treatment, and *γ*_2 _the log-odds ratio of efficacy associated to treatment 2 *versus *the reference treatment.

The center effects are modeled by *γ*_0*i *_where *γ*_01 _= *0 *for identifiability constraint.

##### Testing the proportional odds assumptions

It is worth noting that model (2) assumes that, within each center and for each outcome category *c*, (*c *= 1, .., *m*-1), there is a common log-odds ratio, *γ*_1 _for treatment *l versus *the reference treatment. Similarly, model (2) assumes also that, within each treatment group, and for each outcome category *c *there is a common log-odds ratio, *γ*_0*i *_- *γ*_0*i*_, between two centers *i *and *i'*, independent of the outcome category.

Additional models were introduced to test the PO assumption. Model (3) allows for testing a global PO between treatments, without a center effect:(3)

However, each treatment can have a different effect according to the outcome category.

Assuming a global PO for *T*_1_, the model (4) allows for testing PO between ***T***_2 _and the reference treatment(4)

where *δ*_*hc *_= 1 if *h *= *c *and 0, otherwise.

Similarly, the model (5) assumes a global PO for ***T***_2_, and allows for testing PO between T1 and the reference treatment (here AQ)(5)

##### Mixed effects model

Discarding a center effect, and still assuming proportional odds between treatments, 2 random effects were added to the treatment variables.

where: *β*_1*i *_= *γ*_1 _+ *δ*_1*i *_and *β*_2*i *_= *γ*_2 _+ *δ*_2*i*_. Both *δ*_1*i *_and *δ*_2*i *_are normally distributed random effects with expected value 0 and variance ,  respectively.

By grouping separately the fixed and the random-effects, the model can be rewritten as follows:(6)

where , where *G'*, the transposed matrix of *G*, needs to be estimated from the data.

#### Estimation methods

The parameters of the fixed effects models were estimated using three different methods, *i.e. *maximum likelihood (ML), iterative generalized least squares (IGLS) and generalized estimating equations (GEE) [7]. In the IGLS and GEE methods, the ordinal response was considered as a set of correlated binary responses. Thus the proportional odds model is viewed as a member of the multivariate generalized linear models [13].

All these methods were implemented under the free Statistical Software R http://cran.r-project.org/. The various codes for the ML, GEE and IGLS methods are in the additional file 1. Models (3), (4) and (5) were implemented for the test of the proportional odds assumption between centers and treatments. The parameters of the random effects model were estimated using a Bayesian approach for hierarchical models. In the Bayesian paradigm, prior distributions for all unknown parameters *θ *need to be specified. The intercept *α*_*c *_were given non-informative *N*(0,10^4^) priors. Parameters *β*_1*i *_and *β*_2*i *_were assumed to be from *N*(*γ*_1_, *σ*_1_^2^) and *N*(*γ*_2_, *σ*_2_^2^) respectively. We gave *γ*_1_, *γ*_2 _non-informative *N*(0,10^-6^). The difficulty in the Bayesian paradigm is the choice of the *a priori *distribution for the inverse of the variances *σ*_1_^2 ^and *σ*_2_^2^. Several forms of the *a priori *are available. We chose an *a priori *of the form *Gamma*(0.5*η*, 0.5*ηζ*^2^), where *η *and *ζ*^2 ^are either arbitrary chosen or calibrated from the data. Here we chose *η *= 2. The initial estimated values of *ζ*^2 ^(0,007, 0,001) were obtained from our data, by adjusting a generalized linear mixed model. So 1/*σ*_1_^2 ^and 1/*σ*_2_^2 ^were given *Gamma*(1,0.007) and *Gamma*(1,0.001), respectively. All the Bayesian analyses were performed using 3 chains of 50000 samples, out of which the first 25000 were removed to allow for convergence. Thus, each posterior statistic was based on 75000 samples. Finally the 95% credibility interval was obtained for each statistic.

#### Simulations

To assess the performance of the fixed effect methods, we carried out analyses on simulated data based on model (6). The aim was to compare the methods when the treatment covariate was taken into consideration and random effects were introduced to account for center variability. We generated a data set of 3 centers, each containing 100 patients, comparing 3 treatments A, B, C, with equal size. We supposed that the treatment effects were the same in the 3 centers, and were set to be *γ*_1 _= -1 for B comparing to A and, *γ*_2 _= 1 for C comparing to A, respectively. Initial values for *α*_*c *_were *α*_01 _= -2, *α*_02 _= -1, *α*_03 _= 0. The initial value of the matrix *G*' was a diagonal matrix with diagonal elements *σ*_1 _= 0.01 and *σ*_1 _= 0.02, corresponding to the between center deviation.

The choice of the treatment initial values was guided by preliminary results from real data. The choice for the cut-point values was derived from [14].

Cumulative probabilities *Q*_*ijc *_were then obtained using model (6). For a subject *j *in study *i*, with vector of cumulative probabilities (0, *Q*_*ij*1_, *Q*_*ij*2_, *Q*_*ij*3_, 1), the simulated outcome was generated using the quartile range category, to which an uniformly (0,1) distributed random value belonged. To check that the conclusions were not sensitive to the choice of the starting values, we repeated the simulations, for each method, with different initial values. An example of the simulated data set can be found in additional file 2.

For each method, bias and confidence levels were estimated through *N *1000 = replications. For each estimation method, the treatment effect was assessed using the calculated coverage probability and the type I error.

In the Bayesian analysis, model (6) was implemented using the Bayesian Software WinBUGS. A program is provided in the additional file 3, showing how model (6) can be written in BUGS language.

## Results

### Simulated data set

Results are summarized in Table 2. The ML-, IGLS- and GEE- methods yielded similar results. For the detection bias, the ML estimate appeared less biased than the two other methods. Considering their coverage probabilities (*CP *= 1- *β*), all the three approaches appeared to be able to detect a significant treatment effect with a given type I error *α*.

**Table 2:** Simulation results.

			Methods			Bias (*)			1-*β *(*α*)	
**Parameters**	**Real values**	**ML**	**IGLS**	**GEE**	**ML**	**IGLS**	**GEE**	**ML**	**IGLS**	**GEE**

Cut-points										
*α*_1_	-2	-2.149	-2.149	-2.143	0.014	0.007	0.0137			
		(0.236)	(0.237)	(0.237)	(7.34)	(1.98)	(6.76)			
*α*_1_	-1	-1.053	-1.053	-1.040	0.012	0.002	0.0136			
		(0.214)	(0.211)	(0.217)	(7.35)	(1.69)	(7.91)			
*α*_3_	0	-0.106	-0.106	-0.103	0.0055	-0.0043	0.0025			
		(0.206)	(0.204)	(0.207)	(3.57)	(1.6)	(1.56)			

Treatment effects										
*γ*_01_	-1	-1.016	-1.016	-0.941	0.0077	0.0195	0.0098	**0.938**	**0.948**	**0.94**
		(0.242)	(0.240)	(0.248)	(3.22)	(2.42)	(3.75)	(0.014)	(0.013)	(0.015)
*γ*_02_	1	1.078	1.078	1.082	-0.005	-0.0043	-0.0111	**0.943**	**0.943**	**0.945**
		(0.214)	(0.215)	(0.217)	(2.27)	(2.20)	(5.05)	(0.003)	(0.001)	(0.001)

Center effect										
C_1_		0.218	0.218	0.168						
		(0.224)	(0.224)	(0.228)						
C_2_		-0.014	-0.014	0.013						
		(0.228)	(0.226)	(0.230)						

Fixed models. Column 2 corresponds to the real values used for simulating data.

Columns 3-5 correspond to the mean estimates according to different methods with the corresponding standard error estimates in brackets; columns 6-8 correspond to the corresponding absolute and relative biases (*); columns 9-11 corresponds to the coverage probability and, in brackets, the type I error for the treatment effect only.

### Analysis of the Multi-centre trial

#### Results based on treatment and center effect only

Among the *n *= 538 patients from the three centers, 19 patients were lost to follow-up by day 14 and hence could not contribute to the analyses.

Results from the randomized trial are displayed in Table 3 with a general view of the fixed and the Bayesian approach. All the methods showed a significant decrease of the SP effect as compared with AQ. The SP treatment appeared less efficacious than AQ, by all the three fixed methods (ML: *OR *= *e*^*-1.93 *^= 0.14; 95% CI 0.04-0.50; IGLS: *OR *= *e*^*-1.77 *^= 0.17; 95% CI 0.04-0.58; GEE: *OR *= *e*^*-1.80 *^= 0.16; 95% CI 0.04-0.57). The combination AQ-SP was not significantly different by the three approaches. The additional file 4 shows how practically these fixed estimates can be obtained with an example of the different output given in additional file 5.

**Table 3:** summary of parameter estimates using both the fixed effect models and the bayesian approach in the anti-malarial trial.

		Fixed Approach		Bayesian Approach
	ML	IGLS	GEE	WinBUGS
**Category associated cut-points**				
*α*_1_	3.850	3.880	3.665	3.915
	(0.63)	(0.64)	(0.63)	(0.50)
*α*_2_	4.237	3.880	4.060	4.359
	(0.65)	(0.64)	(0.64)	(0.51)
*α*_3_	4.380	4.237	4.203	4.572
	(0.67)	(0.66)	(0.65)	(0.52)

Treatment (Reference: AQ)				
SP/AQ	-1.938*	-1.776*	-1.800 *	-1.760*
	(0.63)	(0.64)	(0.63)	(0.57)
AQ+SP/AQ	0.537	1.797	2.081	1.601
	(0.97)	(1.53)	(1.72)	(1.09)

Center (Reference: Yaoundé)				
Bertoua/Yaoundé	0.196	0.100	0.242	
	(0.51)	(0.55)	(0.55)	
				
Gardoua/Yaoundé	0.460	0.458	0.475	
	(0.53)	(0.59)	(0.57)	

*Treatment effect estimates in presence of a center effect. For each outcome category, the SP treatment was less efficacious than the AQ treatment.

The between center variability was  = 0.023 (95% credibility interval 0.001-0.107) for the SP/AQ treatment, and  = 0.020 (95% credibility interval 0.0001-0.024) for the AS+SP/AQ treatment. The assumption of proportional odds between treatments and centers was tested using the ML method by comparing the change in deviance of the various models (Table 4) to the chi-squared *χ*_1-*α*_^2 ^(2 df) which equals 5.99. The results indicate that the assumption was satisfactory.

**Table 4:** Test of the proportional odds between centers and treatments on the anti-malarial trial data set.

Tests	Model comparisons	ML results Change in deviance
Proportional odds	5 vs. 3	0.10 (2 d.f.)
between SP and AQ		*p *> 0.95
		
Proportional odds	4 vs. 3	2.24 (2 d.f.)
between AQ-SP and AQ		*p *> 0.32
		
Proportional odds	2 vs. 3	0.2 (2 d.f.)
between centers		*p *> 0.90

#### Results after covariates adjustment

Covariates known at the time of inclusion were the parasitaemia, the type of treatment, the gender, the patient's age, the bodyweight (kg), a previous treatment before the study enrollment, the haematocrit and the baseline body temperature. The patient's bodyweight was log-transformed; the initial parasitaemia counts were transformed to normality using a Box-Cox transformation (estimated power = 0.1028, p-value = 0.0056). The baseline haematocrit was categorized into two classes, below (A) and above (B) 25%. The patient's age was also categorized into 3 categories: i) less than 1 year old (A); ii) between 2 and 4 years old (B); iii) more than 5 years old (C).

First, a univariate analysis including each covariate of interest, in addition to the treatment covariates and the centre covariate was performed. Then only covariates with a p-value under 10% were selected for a multivariate analysis. Only the baseline temperature reached a statistical significance (Table 5). Multivariate analysis did not change the magnitude of the treatment effects.

**Table 5:** Effect of individual covariates on the ordinal outcome.

		Univariate			Multivariate	
	Est	SD	Z-value	Est	SD	Z-value
**Global covariates**						
- Treatment (Reference: AQ)						
SP/AQ	-1.776	0.633	2.805	-1.638	0.566	**2.893**
AQ+SP/AQ	1.797	1.533	1.172	1.418	1.128	1.257
						
- Center (Reference: Yaoundé)						
Bert/Ydé	0.100	0.550	0.181			
Gar/Ydé	0.458	0.589	0.777			
						

**Individual covariates**						
- Baseline temperature*	-0.577	0.264	2.18	-0.614	0.266	2.308**
- Fever before inclusion	-0.003	0.070	0.05			
- Parasitemia	-0.072	0.071	1.02			
- Hematocrit Day 0	0.341	0.518	0.74			
- Automedication Y/N	0.014	0.524	0.03			
- Age B/A	0.391	0.511	0.77			
- Age C/A	0.989	0.728	1.35			
- Age (months)	0.010	0.012	0.87			
- Gender M = 1/F = 0	0.279	0.468	0.60			
- Bodyweights*	1.373	0.716	1.91	0.113	0.06	1.91

Univariate analysis of the individual covariate performed in presence of the treatment covariate.

*Only variable with a p-value below 10% were retained for the multivariate analysis. *: p < 0.05

As all the estimated intercept values were strictly positive, the assumption of a logistic distribution for the latent variable could be replaced with an extreme value distribution, using a model with a complementary log-log (*cloglog*) link function [2]. In Table 6, the corresponding results show that the intercept value-estimates were reduced as compared to the corresponding ones using the logistic distribution. However, the treatment parameter estimates did not change although the interpretation is not strictly the same. Indeed, as pointed out by Agresti [2], trying a complementary log-log link function is equivalent to fitting a proportional hazards model. Hence regarding the comparison of the SP treatment to the reference treatment AQ, the parameter *e*^-0.502 ^= 0.605 (95% CI 0.44-0.82) is interpreted as a hazards ratio which is assumed constant regardless the response category. It suggests that the proportion of patients having positive response was more decreased with SP than with AQ. Between AQSP and AQ, the hazards ratio was 1.40 (95% CI 0.88-2.18), suggesting that there was no difference in efficacy.

**Table 6:** Model fitting with the complementary log-log link function and results comparisons.

	ML	ML	ML	ML
	Logit	Cloglog	Logit	Cloglog
Category cut-points				
*α*_1_	3.850	1.297	25.980	8.201
	(0.63)	(0.16)	(10.65)	(3.54)
*α*_2_	4.237	1.431	26.39	8.346
	(0.65)	(0.17)	(10.66)	(3.54)
*α*_3_	4.380	1.478	26.540	8.401
	(0.6¬¬6)	(0.17)	(10.66)	(3.54)

Treatment (Reference AQ)				
SP/AQ	-1.938	-0.524	-1.638	-0.502
	(0.63)	(0.16)	(0.57)	(0.16)
AQ+SP/AQ	0.537	0.348	1.416	0.329
	(0.97)	(0.24)	(1.13)	(0.23)

Center (Reference Yaoundé)				
Bert/Ydé	0.196	-0.016		
	(0.51)	(0.17)		
Gar/Ydé	0.460	0.123		
	(0.53)	(0.18)		

Individual Covariate				
Baseline temperature	-0.604	0.188		
	(0.27)	(0.09)		
Bodyweights	0.113	0.037		
	(0.06)	(0.02)		

Parameter estimates with the corresponding standard error estimates on the following line.

## Discussion

The present work applied methods, initially presented in the context of a meta-analysis comparing 2 treatment effects with a categorical outcome, to the analysis of multi-center-antimalarial trials, comparing 3 treatments, with a 4-categorical primary outcome, using individual patient data. All the methods were implemented using free available softwares. Their performances were assessed using simulated data sets. Both a frequentist and Bayesian approaches were implemented. In the simulation settings, we considered a general case of a mixed effects model. The ML method was faster than the IGLS and GEE methods. All the methods produced minimal biases. Another R function can be used like *lrm *(*Hmisc *library using the library *Design*) which is more restricted at present in the choice of the link functions.

Our results from the multi-centre trial showed a significant decrease in efficacy in the *sulfadoxine-pyrimethamine*-arm as compared with the *amodiaquine*-arm. This means that there was less ACPR and more failures with SP. No significant difference was observed between amodiaquine and the combination AQ + SP. Similar results hold for both the frequentist and Bayesian approaches. The proportional odds assumption between treatments was not rejected. The proposed model can be a useful way when dealing with a small number of centers with more than 2 treatments.

Regarding the dependence between the outcome and the explanatory variables, a recent study [15] reported patient's age as a significant factor associated with antimalarial treatment failure, more specifically with artemisinin derivatives and quinine. Indeed, the study showed that children less than 5 years old had a significant risk of treatment failure as compared with children more than 5 years. In our analysis, no significant age effect was found, but our data concerned mostly children less than 5 years. Covariates, like haematocrit, gender, auto-medication before study enrollment, were not significant. In contrast, the baseline temperature was significantly associated with the outcome, showing that patients with a low temperature at inclusion were more likely to have a good treatment response. For the SP treatment, the decrease could be related to a gene mutation, responsible for a resistance towards SP [16-18]. The SP resistance was also confirmed in a recent study in South Africa [19]. The emergence of resistance led to the use of Artemisinin-based Combination Therapies (ACTs) which play an important role in the reduction of malaria transmission [20]. Thus, ACTs are widely used as first-line treatment of malaria in Africa [21-28].

The present work presented some statistical methods to handle the WHO criteria globally. They appear adapted for assessing antimalarial treatment in clinical trials, or in other studies with an ordinal outcome. It contrasts with previous methods [15], which reduced the outcome into a binary variable, for instance ACPR *versus *non ACPR. Only 3.5% were missing data. By eliminating drop-out or excluded patients in the final analysis, we assumed that they were missing completely at random (MCAR). Incorporating these into the analysis would require an imputation of the missing category. For such imputation, wide-range methods have been studied [29], but remain a topic to study in the case of malaria.

Other study designs are presently available in which patients have repeated standardized visits during their follow-up. Thus repeated categorical responses are available for each subject. All methods used in the case of a single categorical response, can however, be extended to repeated categorical responses [30].

## Conclusion

The approach of modeling the WHO criterion as an ordinal criterion is innovative in the sense that, it gives a gain of information and precision on the estimate of the treatment effect using a global analysis of the four-category outcome instead of either comparing the rate of ACPR only, without being able to differentiate treatments when they present an identical percentages in the ACPR category but, different repartitions in the remaining three other categories, or repeating a similar binary analysis for each category, then exposing to the increase of the type-1 error. This analysis could help in avoiding a conclusion on an equal treatment effect whereas there is not or, vice-versa. In addition, this approach is able to take into account individual's covariates to improve the prediction of efficacy.

## Abbreviations

OCEAC: organisation de coordination pour la lutte contre les endémies en Afrique Centrale; IRD: institut de recherche pour le développement; SP: sulphadoxine-pyrimethamine; AQ: amodiaquine.

## Competing interests

The authors declare that they have no competing interests.

## Authors' contributions

SYW developed the analysis plan and carried out both the statistical analyses and software implementations under the close supervision of HG and JCT. She also drafted the manuscript. LKB was responsible for the overall data collection. All authors read and approved the final manuscript.

## Pre-publication history

The pre-publication history for this paper can be accessed here:

http://www.biomedcentral.com/1471-2288/10/58/prepub

## Supplementary Material

Additional file 1 **RUtilities**. Some utility functions for the fixed effect model using R codes. Click here for file

Additional file 2 **Data**. The simulated data set on which we illustrated the different fixed effects methods. It contains 14 columns whose descriptions are detailed in the additional file 5. Click here for file

Additional file 3 **MixedModelWinBUGSCode**. WinBUGS code for the mixed effects model. To be run after having installed the WinBUGS 1.4 free software downloadable at http://www.mrc-bsu.cam.ac.uk/bugs/winbugs/contents.shtml. Click here for file

Additional file 4 **FixedModelRCode**. This file contains a code to estimate the different mixed effect models. They were run under the software R version 2.5. The order of execution is given in the corresponding file. Click here for file

Additional file 5 **ExampleOutputFile**. This file contains the description of the simulated data set. It provides some outputs of the programs written in the additional file 4. Click here for file
